# A sense of proximity: Cell packing modulates oxygen consumption

**DOI:** 10.1063/5.0160422

**Published:** 2023-08-29

**Authors:** Ermes Botte, Piera Mancini, Chiara Magliaro, Arti Ahluwalia

**Affiliations:** 1 Research Centre “E. Piaggio,” University of Pisa, Pisa, Italy; 2 Department of Information Engineering, University of Pisa, Pisa, Italy

## Abstract

Accurately modeling oxygen transport and consumption is crucial to predict metabolic dynamics in cell cultures and optimize the design of tissue and organ models. We present a methodology to characterize the Michaelis–Menten oxygen consumption parameters *in vitro*, integrating novel experimental techniques and computational tools. The parameters were derived for hepatic cell cultures with different dimensionality (i.e., 2D and 3D) and with different surface and volumetric densities. To quantify cell packing regardless of the dimensionality of cultures, we devised an image-based metric, referred to as the proximity index. The Michaelis–Menten parameters were related to the proximity index through an uptake coefficient, analogous to a diffusion constant, enabling the quantitative analysis of oxygen dynamics across dimensions. Our results show that Michaelis–Menten parameters are not constant for a given cell type but change with dimensionality and cell density. The maximum consumption rate per cell decreases significantly with cell surface and volumetric density, while the Michaelis–Menten constant tends to increase. In addition, the dependency of the uptake coefficient on the proximity index suggests that the oxygen consumption rate of hepatic cells is superadaptive, as they modulate their oxygen utilization according to its local availability and to the proximity of other cells. We describe, for the first time, how cells consume oxygen as a function of cell proximity, through a quantitative index, which combines cell density and dimensionality. This study enhances our understanding of how cell–cell interaction affects oxygen dynamics and enables better prediction of aerobic metabolism in tissue models, improving their translational value.

## INTRODUCTION

Oxygen (O_2_) is a key player in cellular respiration. Its consumption rate (i.e., metabolism) is one of the major determinants of metabolic activity, which in turn strongly influences cell growth and fate.^1^ O_2_'s low solubility in aqueous media results in an inadequate supply of this primary energy resource to cells, particularly in high-density,three-dimensional (3D) tissue constructs, which are known to form hypoxic cores. This is recognized as one of the factors limiting the prospects of tissue and organ models *in vitro.*^2–6^

Investigating O_2_ metabolism in cellular models—be they *in silico* or *in vitro*—is not straightforward because it depends on both intrinsic and extrinsic factors. For instance, mammalian cells have been widely reported to display different metabolic behavior depending on their microenvironment.^7–11^ The size or extent of cell cultures, their density, and spatial arrangement in two or three dimensions (i.e., the dimensionality), as well as structural and functional characteristics of the substrate are also known to have a major impact on cellular activity.^12,13^ Nonetheless, few reports focus on the identification of quantitative aspects of O_2_ metabolism *in vitro*, and even fewer consider or attempt to control the different variables known to be involved in the modulation of cellular O_2_ consumption.^14–16^

A widely accepted analytical formulation for cellular O_2_ consumption kinetics is the Michaelis–Menten (MM) model,^4,17,18^ which is fully described by two parameters—the maximum single-cell O_2_ consumption rate ( sOCR, in mol s^−1^) and the MM constant ( $k_{M}$, in mol m^−3^). The MM can be used to express the O_2_ consumption rate ( $R_{\text{cell}}$, in mol s^−1^) for a single cell as follows:

$$R_{\text{cell}}=-\mathrm{sOCR}\frac{C}{k_{M}+C},$$ (1)

where C (mol m^−3^) is the O_2_ concentration perceived by the cell. Thus, the cellular consumption rate is adaptive with respect to ambient O_2_: at high O_2_ levels ( C ≫ $k_{M}$) a cell consumes at its maximal rate (i.e., $R_{\text{cell}}\;≅-\mathrm{sOCR}$), while when resource availability is low ( C ≪ $k_{M}$), the rate of consumption decreases proportionately (i.e., $R_{\text{cell}}=-\frac{\mathrm{sOCR}}{k_{M}}C$).

One of the best-known variables that conditions O_2_ availability and, hence, consumption rate is the height of cell culture media in dishes, plates, or wells. Some theoretical studies and, as far as we know, only one experimental investigation^19^ have sought to demonstrate the dependence of O_2_ metabolism on the level of medium. It should also be noted that, although the MM kinetic parameters are often intended as intrinsic features of the specific cell phenotype, recent reports show that they are likely to vary with experimental conditions (e.g., cell density),^20^ with direct implications on aerobic metabolism and, thus, cell and tissue function, as well as on related biophysical phenomena, such as size-dependent metabolic scaling.^21^ Nonetheless, most investigations on *in vitro* cultures disregard MM kinetics and consider cellular O_2_ consumption rates to be constant and independent of differing ambient O_2_ levels or other factors.^10,22^

The quest to engineer physiologically relevant models—that is, models which better recapitulate the behavior and function of *in vivo* tissues and organs—has propelled the use of 3D culture systems, such as scaffolds, spheroids, organoids, and microtissues. This in turn necessitates the design of constructs with adequate levels of O_2_ supply to all cells, requiring more accurate tools and methods to quantify and therefore predict O_2_ consumption *in vitro*. Do cells change their kinetic parameters when interacting with neighboring cells? Does the number of neighbors and their vicinity modulate their metabolic behavior? In short, can cells sense their local dimension, and can we exploit this knowledge to develop more physiologically relevant and, thus, translatable *in vitro* systems?

**FIG. 1.**
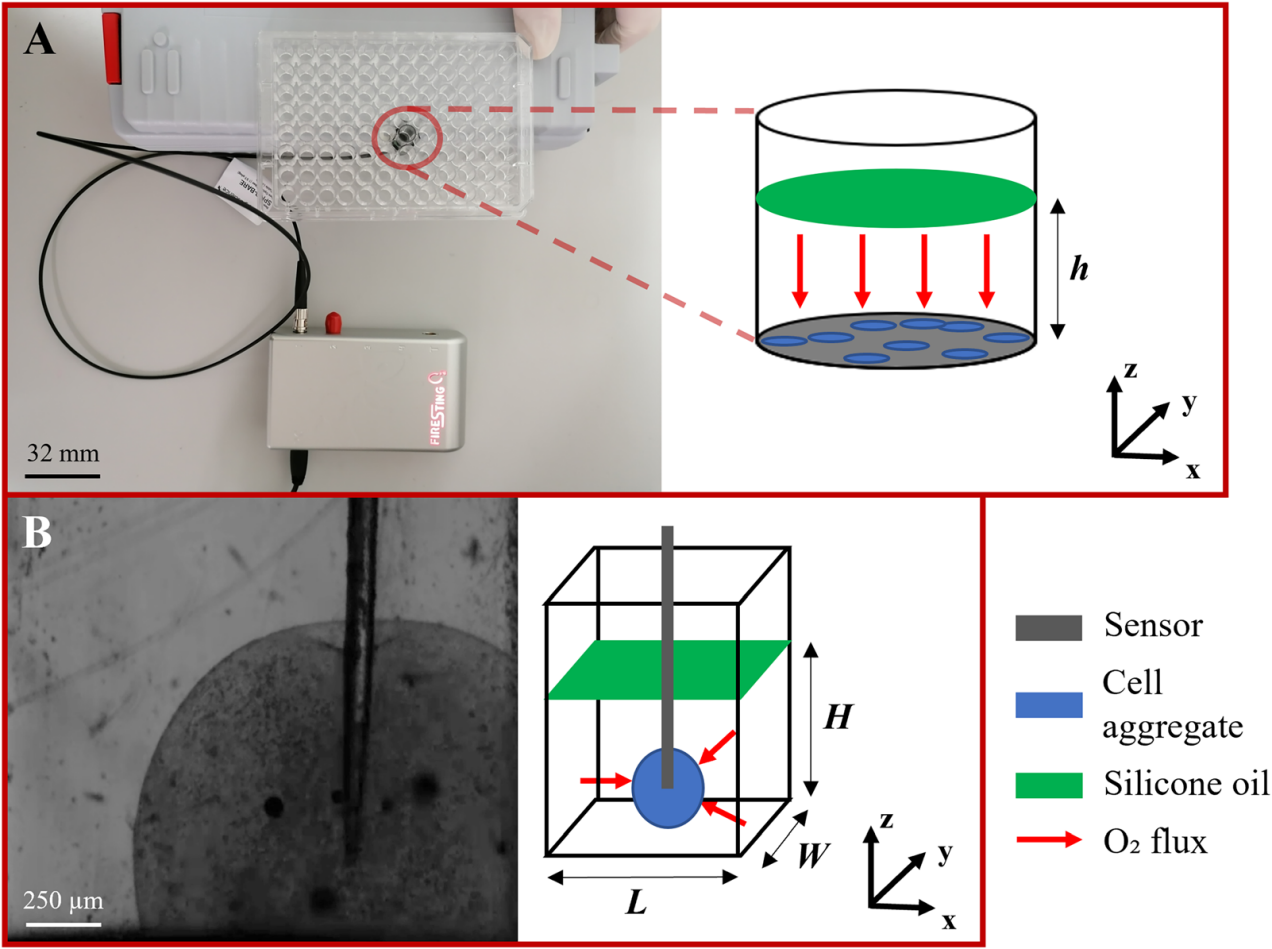
O_2_ sensing setup for the monolayer and 3D configurations. (A) The 96-well plate containing the O_2_ sensor spot where HepG2 monolayers were cultured. (B) HepG2-laden spheroid punctured with the O_2_ microsensor, frontal view through a transparent cuvette. A schematic representation highlighting the main components and O_2_ transport directions is reported for both configurations.

To address these questions, we designed an integrated approach to study the O_2_ consumption characteristics of a human hepatic cell line, HepG2,^23^ using the MM equation as a starting point. More in detail, using the experimental set-up in Fig. 1, O_2_ concentration measurements recorded over time in two-dimensional (2D) monolayer cultures and 3D cell-laden spheroids were analyzed to extract reliable values of sOCR and $k_{M}$ through a purposely developed multiparameter identification algorithm. The MM parameters were then related to the cell density and dimensionality, quantified by means of imaging-based metrics. Using this approach, we show that kinetic parameters are not pre-determined but context-dependent, and this superadaptive behavior may underlie the emergence of cooperative and, hence, physiologically relevant behavior in specific culture conditions.

## RESULTS

### MM parameters and cell density

**FIG. 2 .**
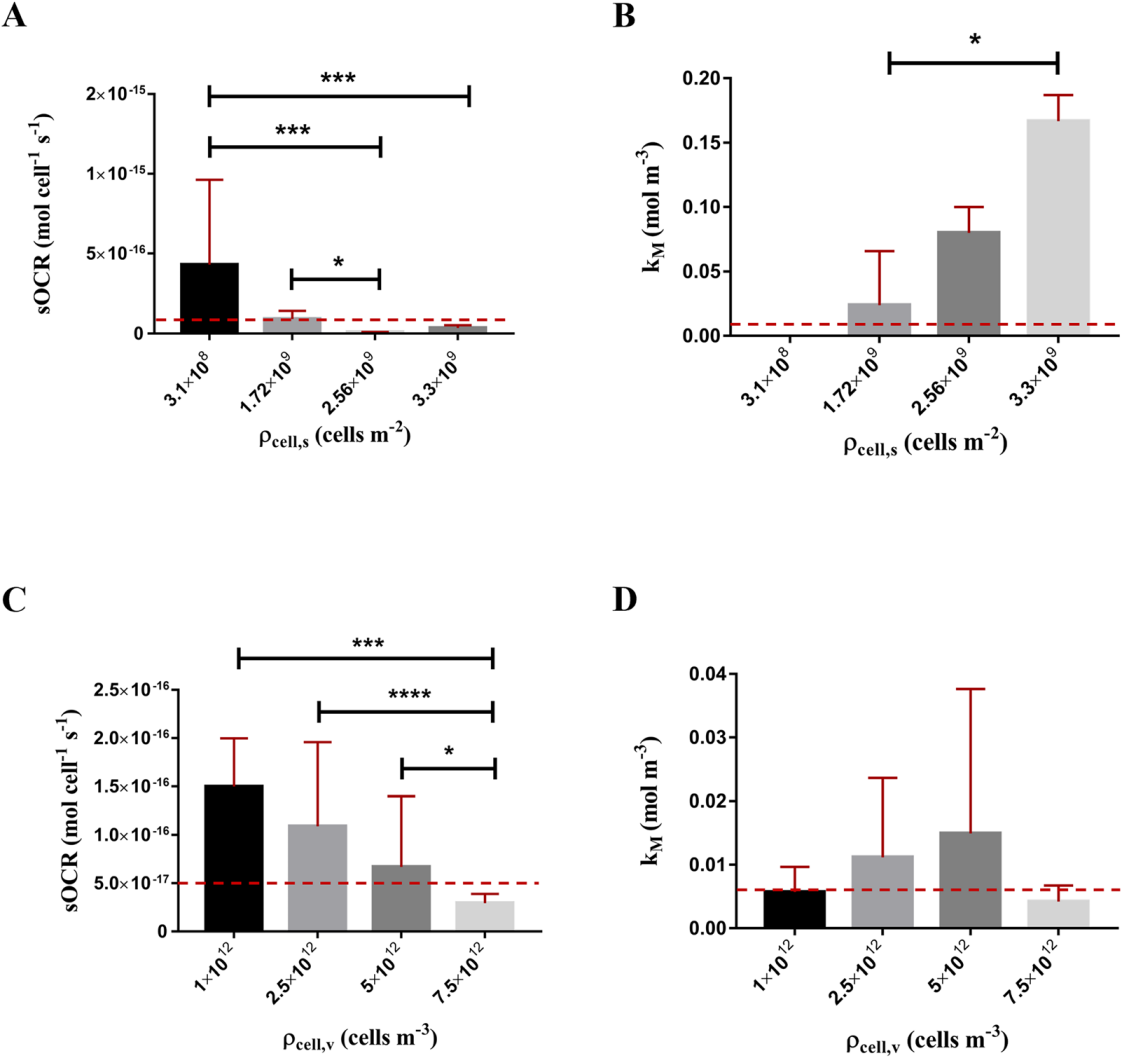
MM parameters identified for monolayers (A) and (B) and cell-laden spheroids (C) and (D). In (B), $k_{M}\;\to$ 0 for $\rho_{\text{cell},s}$ = 3.1 × 10^8^ cells m^−2^ as it was not identifiable. Pairwise statistical differences through *post hoc* Dunn's multiple comparisons (^*^ *p* < 0.05, ^***^ *p* < 0.0005, and ^****^ *p* < 0.0001). Red dashed lines indicate sOCR and $k_{M}$ values from the literature (referring to $\rho_{\text{cell},s}\;\in$ [1.6; 31.2]×10^8^ cells m^−2^ in (A) and (B), and $\rho_{\text{cell},v}\;\in$ [0.5; 50] ×10^12^ in (C) and (D).^7,20,24–26^ All data are expressed as median ± range.

The values of sOCR and $k_{M}$ identified for different surface cell densities of monolayers are shown in Figs. 2(A) and 2(B), respectively. For both parameters, the Kruskal–Wallis test highlighted that medians are not constant across densities (*p* < 0.0001 and *p* = 0.002 for sOCR and $k_{M}$, respectively). sOCR decreases with the surface cell density [Fig. 2(A), Spearman coefficient *r* = −0.8], while $k_{M}$ increases significantly [Fig. 2(B), *r* = 1]. Note that $k_{M}$ values were not identifiable for the lowest cell density considered in the study, since the number of seeded cells was too low to guarantee a stable hypoxic condition, implying that $k_{M}$ is below the limit of sensitivity of our measurement setup. Hence, it was assumed equal to zero [Fig. 2(B)], that is, cells in the monolayer consume O_2_ according to a zero-order kinetics independent of ambient O_2_ levels. The values of consumption parameters identified for HepG2 monolayers are in line with previous estimations in the literature, especially for the lower surface cell densities.^7,20,24–26^ At higher densities—higher than those typically used in most experiments—we observe a notable increase in $k_{M}$, which might indicate metabolic cooperation whereby the cells self-limit their consumption rate. Magliaro *et al.* report a similar trend in cylindrical cell-laden hydrogels.^20^

The dependency of sOCR on the volumetric cell density of spheroids reported in Fig. 2(C) could be interpreted in the same light. In fact, statistical differences emerging through the Kruskal–Wallis test (*p* < 0.0001) and the associated negative correlation with respect to volumetric cell densities (*r* = −0.8) suggest cooperative behavior in terms of O_2_ consumption, analogous to that noticed for 2D cultures [Fig. 2(D)]. Neither significant differences among medians (*p* = 0.9030) nor correlations (*r* = −0.2) were observed for $k_{M}$. However, the median values of $k_{M}$ tend to increase for cell densities up to 5 × 10^12^ cell m^−3^ [Fig. 2(D)], as might be expected for cooperative behavior.

It is worth noting that the dataset obtained for $k_{M}$ is characterized by a large dispersion, probably associated with the relatively small number of tested samples as well as unavoidable experimental errors. The estimation of $k_{M}$ is particularly sensitive to the latter because its value is often close to sensing limits.^27,28^ Such factors may mask differences, if any, related to cell density. We should also point out that the low $k_{M}$ values identified for the densest spheroids are likely to be an artifact due to the reduced cell viability that we noted (the fraction of viable cells was consistently ∼90% compared with ≥ 98% for the other densities). Taken together, our data show a modulation of resource uptake driven by cooperation when O_2_ depletion occurs in densely populated spheroids—statistically confirmed only for sOCR. Again, the kinetic parameters identified approach values reported for 3D hepatocyte cultures in the literature.^7,20,24–26^

Concerning the mono-parametric estimation of MM parameters, reproducible estimations of $k_{M}$ were returned for some literature values of sOCR (data not shown). On the other hand, the algorithm generated widely dispersed and inconsistent estimations of sOCR or was even unable to identify this parameter within the threshold of tolerance if $k_{M}$ was fixed. This confirms that a global optimization approach like ours is necessary to properly estimate both MM parameters and further underlines the challenge of measuring $k_{M}$.

### Dimensionality and sphericity

**FIG. 3.**
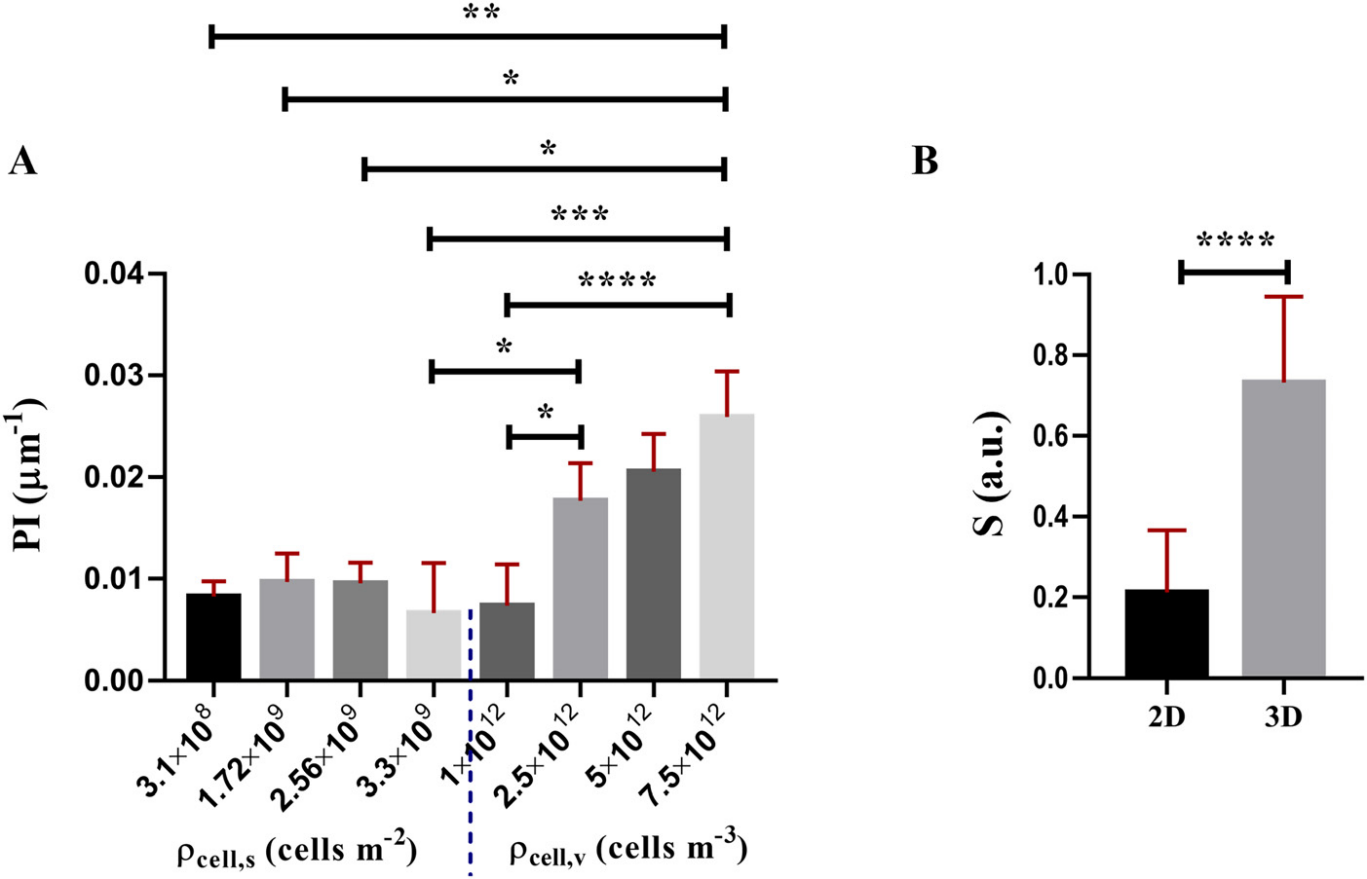
(A) PI values estimated for each surface and volumetric cell density. (B) Nucleus sphericity of single cells cultured in monolayers (2D) and spheroids (3D). All data are reported as median ± range. ^*^ *p* < 0.05, ^**^ *p* < 0.005, ^***^ *p* < 0.0005, and ^****^ *p* < 0.0001.

To identify a common index to describe the extent of cell packing independent of the culture dimensionality, we extracted a proximity index, *PI*, through image processing of confocal stacks [Eq. (6)]. Figure 3(A) shows PI values for the different surface and volumetric cell densities. Pairwise Dunn's comparisons resulted in a basically constant PI for all surface cell densities in the monolayers, while it increases notably with volumetric density for 3D cultures. Furthermore, the PI value of the monolayers was significantly lower than those obtained for spheroids. Irrespective of the slicing orientation, no relevant variations were observed when computing the PI on 10 *μ*m cross-sectional slices in 3D, suggesting that the extent of packing can be reasonably assumed homogeneous within the same cell culture.

Nucleus sphericity was significantly different for the two dimensionalities investigated but did not depend on their density. Thus, data from all monolayers and spheroids were (respectively) lumped together. In coherence with the PI, the sphericity determined for the nuclei of HepG2 cells was significantly lower in 2D than in 3D [Fig. 3(B)]. While it is well-known that cells plated in monolayers tend to spread adopting a flatter shape than in 3D, our results suggest that the extent of flattening is irrespective of surface density. Similarly, cells encapsulated in the hydrogel matrix maintain a rounder shape and this roundness does not depend on the number of cells per unit volume.

### Finding a common metric

We further introduced an *uptake coefficient* φ=sOCR PIkM (μm^2^ s^-1^), which can be interpreted as the area around a cell through which the O_2_ it consumes passes per unit time. It expresses an analog of *D* for consumption and depends on the extent of cell packing (i.e., the combination of dimensionality and cell density) of the culture; the higher its value, the greater the area available to a cell. Thus, a dimensionless group expressing the local balance between φ and *D*, which we refer to as the local supply-to-uptake ratio (*LSU* = φ*/D*) can be defined. The *LSU* is conceptually similar to the Thiele modulus, since it represents the ratio between the O_2_ consumption and diffusion behavior of the culture, irrespective of its size, density, and dimensionality. The dependence of O_2_ consumption kinetics on the *PI*, and the derived parameters, φ and *LSU*, was investigated. Although statistical differences among medians for both sOCR and $k_{M}$ with respect to PI were observed, there were no significant trends or correlations. Refer to the supplementary material (Sec. SM5 and Fig. S3) for further details in this regard.

**FIG. 4.**
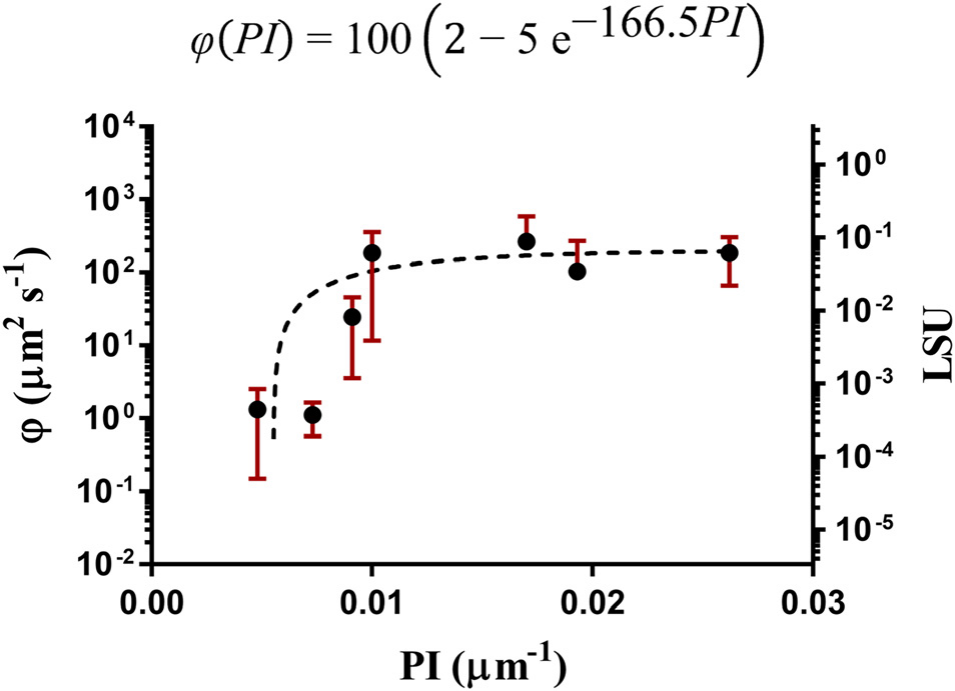
Outcome of the multiparameter identification procedure expressed through the uptake coefficient ( φ, left vertical axis) and the local supply-to-uptake ratio ( LSU, right vertical axis) as a function of the PI. An arbitrary exponential fit of φ as a function of PI (black dashed curve, *R*^2^ = 0.5698) is reported. All data are expressed as median ± range, computed from measured values of sOCR, $k_{M}$, and PI by applying standard rules for error propagation. Datapoints were fitted weighting by the corresponding extents of dispersion. For the sake of visualization, the vertical axes are in logarithmic scale. Other fittings are reported in the supplementary material (Sec. SM6).

However, combining sOCR and $k_{M}$ to compute the uptake coefficient φ, we noticed a significant correlation between φ and corresponding PI values (*r* = 0.7857, Fig. 4). After a steep raise for cell cultures with the lowest values of PI, the coefficient φ saturates. An arbitrary exponential equation reasonably fits the φ vs PI data (*R*^2^ = 0.5698), as reported in Fig. 4. Since φ represents a measure of the surface per second related to O_2_ uptake, this trend indicates that the ability of cells to recruit O_2_ from the surroundings increases rapidly when the cells are far apart (irrespective of dimensionality) but tends to a constant when they are tightly packed. Sigmoidal and Langmuir-type equations were also tested but resulted in weaker statistical significance (see supplementary material, Sec. SM6).

LSU is < 0.1 across all PI values, indicating that the diffusion locally remains faster than consumption even in highly populated cell cultures. In coherence with previous observations, this result suggests that when cells are close to their neighbors, they modulate their O_2_ uptake to maintain a balance with respect to the diffusive supply.

## DISCUSSION

Here, we demonstrate a novel method for systematically characterizing the O_2_ consumption kinetics of cells across different dimensionalities and densities, starting from the well-known MM formulation. Our results underline the fundamental role of cooperation in shaping the O_2_ metabolism of cell cultures.^29,30^ In fact, for all cultures, the values of sOCR and $k_{M}$ identified as well as the trends observed indicate that O_2_ uptake is modulated as a function of (surface or volumetric) cell density (Fig. 2).

However, the contribution of dimensionality is impossible to infer unless a common metric that combines cell density and spatial arrangement can be identified. Here, we introduce a new imaging-based index—the so-called PI—for quantifying the closeness of cells regardless of their dimensionality [Eqs. (5) and (6)]. Based on this unique index, an uptake coefficient φ, which combines the experimentally derived MM parameters and PI, was defined. φ synthetically describes the ability of cells to “sense” their neighbors and, thus, modulate their O_2_ uptake. In particular, φ increases with PI for poorly packed cultures, up to a saturation level for the highest PI values (Fig. 4). Consequently, a proper supply-to-uptake equilibrium is locally guaranteed. We also introduced the LSU, a dimensionless number, which represents the ratio between φ and D. Similar to Thiele's modulus, it quantifies the competing contributions of O_2_ consumption and diffusion dynamics at the cellular level. We observed that LSU *<* 0.1 (Fig. 4), even in conditions of maximal packing, indicating that diffusion is faster than consumption.

In this study, we used HepG2 monolayers and HepG2-laden spheroids, measuring local O_2_ consumption rates with microprobes or patches. While our data are in agreement with literature values for similar systems, the sOCR reported here differs significantly from maximal O_2_ consumption rates per cell measured for perfused *in vitro* hepatic microtissues^22^ and *ex vivo* rat and mouse livers^31–34^ (our sOCR is about three orders of magnitude lower). However, these studies refer to hepatic tissues—be they engineered or from animal models—containing a variety of cell types present in the liver and rely on the measurement of O_2_ concentration differences between the inlet and outlet of the perfusion circuit rather than pointwise monitoring of the O_2_ level over time. Moreover, most reports^22,31,32^ model O_2_ consumption using zero-order kinetics, neglecting adaptation to local availability as described by MM kinetics. Despite these differences, where available, the half saturation constants estimated for perfused rat and mouse livers (∼0.02 mol m^−3^) match our $k_{M}$ values, particularly for the higher volumetric densities.

It is well known that cells behave differently in 2- and 3D and that cell–cell interaction is fundamental to their function. In this study, we sought to identify a single metric capable of describing the volumetric O_2_ consumption rate of hepatic cells as a function of their dimensionality and extent of cell–cell vicinity. The dependence of parameter φ—and the corresponding dimensionless LSU—on the proximity index PI suggests that the metabolic behavior of the cells depends only on consumption parameters and the extent of cell packing rather than on culture conditions (e.g*.,* O_2_ availability, dimensionality). Although further investigations—more experimental repeats covering a wider range of densities and the use of different cell phenotypes—are necessary to verify the statistical significance and generalizability of our data, this study demonstrates the importance of considering the O_2_ consumption rate of a cell culture in terms of phenomena such as cell adaptation, cell–cell cooperation, and coexistence.^35^ These phenomena require a superadaptive kinetic formulation and cannot be explicitly described by means of the MM model, which only considers the dependence of consumption rate on the local O_2_ concentration.

## CONCLUSION

A quantitative description of O_2_ metabolism in cell cultures is crucial for designing physiologically relevant *in vitro* models. In this work, we measured cellular O_2_ consumption and linked it to a quantitative descriptor of cell packing. Our results suggest that cells in culture adopt adaptive mechanisms that depend on the proximity of their neighbors. To account for this effect, we refined the current formulation of O_2_ consumption kinetics, exploiting the observed dependency of φ (or, equivalently, LSU) on the extent of cell packing, which we describe through the PI. If corroborated by additional data, a general relationship, such as that depicted in Fig. 4, could be used to express $k_{M}$ as a function of sOCR and the PI. Bearing in mind the challenge of accurately identifying $k_{M}$ as discussed previously, this would represent a fundamental improvement in the characterization of O_2_ consumption kinetics. In particular, measuring maximal rates of consumption (i.e., sOCR) in O_2_-saturated conditions and estimating the PI of cell cultures using well-established imaging techniques would be more feasible and reproducible than attempting to monitor O_2_ metabolism in stable hypoxia. Further studies to elucidate the biochemical mechanisms underlying the observed superadaptive behavior could help better understand why *sOCR* and *k_M_* depend on the average proximity of cells. In fact, linking the modulation of MM parameters to the underlying biochemical pathways (e.g., hypoxia inducible factor-related mechanisms) could contribute to the development of a more accurate mathematical formulation of O_2_ consumption kinetics in cell cultures, which implicitly includes the dependency on cell proximity. Finally, a more exhaustive description of cell superadaptivity to both local O_2_ concentration and cell packing might have implications beyond tissue engineering, from life sciences to metabolic regulation.

## METHODS

### The mathematical model

Analytically speaking, measuring kinetic parameters requires monitoring the O_2_ concentration field $C=C\left(x,\;y,\;x,\;t\right)$ over time in a region of the space with a fixed volume, where the generalized second Fick's law [Eq. (2)] holds,

$$\frac{\partial C}{\partial t}=D\nabla^{2}C+R,$$ (2)

where $R=R\left(x,\;y,\;z,\;t\right)$ (mol m^−3^ s^−1^) is the O_2_ production/consumption rate per unit volume of interest, and D (m^2^ s^−1^) is the diffusion constant of O_2_ in water. In the case of consumption, R is negative and, as outlined in the introduction, typically described via the MM model. Thus,

$$R=-\rho_{\text{cell},v}s\mathrm{OCR}\frac{C}{k_{M}+C},$$ (3)

where $\rho_{\text{cell},v}$ (cell m^−3^) is the cell density in the volume, and sOCR and $k_{M}$ are the MM parameters to be identified. Note that Eq. (3) assumes that sOCR and $k_{M}$ are constants, that is, each cell in the volume considered has the same metabolic sensitivity to O_2_ levels and consumes at the same maximal rate, and that the cells are homogenously distributed in the volume.

In the case of monolayers, assuming that cells are homogeneously distributed over a plane (e.g*.,* the bottom of a culture dish), O_2_ dynamics is only dependent on the axial direction [i.e., $C=C\left(z,\;t\right)$, see Fig. 1(A)]. Then, neglecting cell thickness, O_2_ consumption can be modeled as a surface reaction, physically equivalent to an inward O_2_ flux (Table I). Thus, Eq. (2) can be written as

$$\frac{\partial C}{\partial t}=D\frac{\partial^{2}C}{\partial z^{2}}.$$ (4)

**TABLE I.** Initial and boundary conditions implemented for simulating O_2_ dynamics for both dimensionalities (cell monolayers = 2D, cell-laden spheroids = 3D). n denotes the pointwise normal direction to the considered boundary.

Dimensionality	Boundary	Condition	Description
2D	Air–medium interface	$n\cdot J\left(h,t\right)=0$	No flux
2D	Well bottom	$n\cdot J\left(0,t\right)=-\rho_{\text{cell},s}s\mathrm{OCR}\frac{C}{k_{M}+C}$	Surface consumption
3D	Air–medium interface, cuvette walls	n·J±L2,y,z,t=0n·Jx,±W2,z,t=0n·Jx,y,0,t=0n·Jx,y,H,t=0	No flux
3D	Spheroid surface	$n\cdot \left(J_{\text{med}}\left(\bar{x},\bar{y},\bar{z},t\right)-J_{\text{sph}}\left(\bar{x},\bar{y},\bar{z},t\right)\right)=0$	Continuity

### The challenge of measuring *sOCR* and *k_M_*

Several practical considerations need to be considered to estimate the MM parameters for O_2_ metabolism. Obviously, the sensor must have a response time, which is much faster than the kinetics being monitored and possess adequate sensitivity to changes in O_2_ levels. Furthermore, to determine sOCR and $k_{M}$, the cells must be probed over a range of O_2_ concentrations. Specifically, to accurately estimate $k_{M}$, they need to be exposed to 100% O_2_ saturation conditions down to concentrations much less than $k_{M}$. As $k_{M}$ is often much lower than ambient O_2_, this requires the cells to be brought to near anoxia. To achieve this condition, great care needs to be taken to ensure that the system is impermeable to atmospheric O_2_. We found that the standard method for achieving this (i.e., a layer of silicone oil atop the medium) is not sufficient, particularly when R is high. Thus, additional precautions for reducing O_2_ diffusion at the air–media interface were purposely implemented as described herein.

Additionally, in 3D constructs, the rate of change of O_2_ concentration varies in space and time, as expressed in Eq. (2). Hence, it must be locally monitored over time with high spatial resolution. Specifically, measuring changes in concentration over time requires a sensor with dimensions that are negligible in comparison to the volume of interest, and its positioning must be accurately controlled and known during experiments. On the other hand, 2D cultures require sensors able to measure O_2_ levels in the vicinity of a surface. Finally, to make appropriate comparisons as a function of dimensionality, the sensors should ideally exploit the same physical principle.

### Cell preparation

HepG2 cells are a human hepatocellular carcinoma cell line (ATCC^®^, Manassas, Virginia, USA). While they lack drug-metabolizing enzymes, their endogenous metabolism is largely intact.^36^ They were cultured as described in the supplementary material (Sec. SM1); then, 2D and 3D cell aggregates were prepared as follows.

For monolayers, before cell seeding, the surface of the chosen flat sensor (see the sub-section “Oxygen concentration measurements”) was coated with a 1% w/v alginic acid (Sigma-Aldrich^®^, St Louis, Missouri, USA) solution containing 0.004% type I collagen (Life Technologies Corporation^®^, New York, USA), followed by an overlaying volume of 20 *μ*l of 0.1 M calcium chloride (CaCl_2_) for gelation. After 15 min of incubation, the CaCl_2_ solution was removed, and the surface of the hydrogel layer rinsed with phosphate buffered saline (PBS, Lonza^®^) 1×. Then, HepG2 cells were suspended in 200 *μ*l of Dulbecco's modified Eagle medium (DMEM, Sigma-Aldrich^®^), dispensed onto the prepared sensor and incubated overnight (37 °C, 5% CO_2_, 95% RH), so as to obtain surface densities ranging from 3.10 × 10^8^ to 3.30 × 10^9^ cell m^−2^, as reported in the supplementary material (Table S1).

**FIG. 5.**
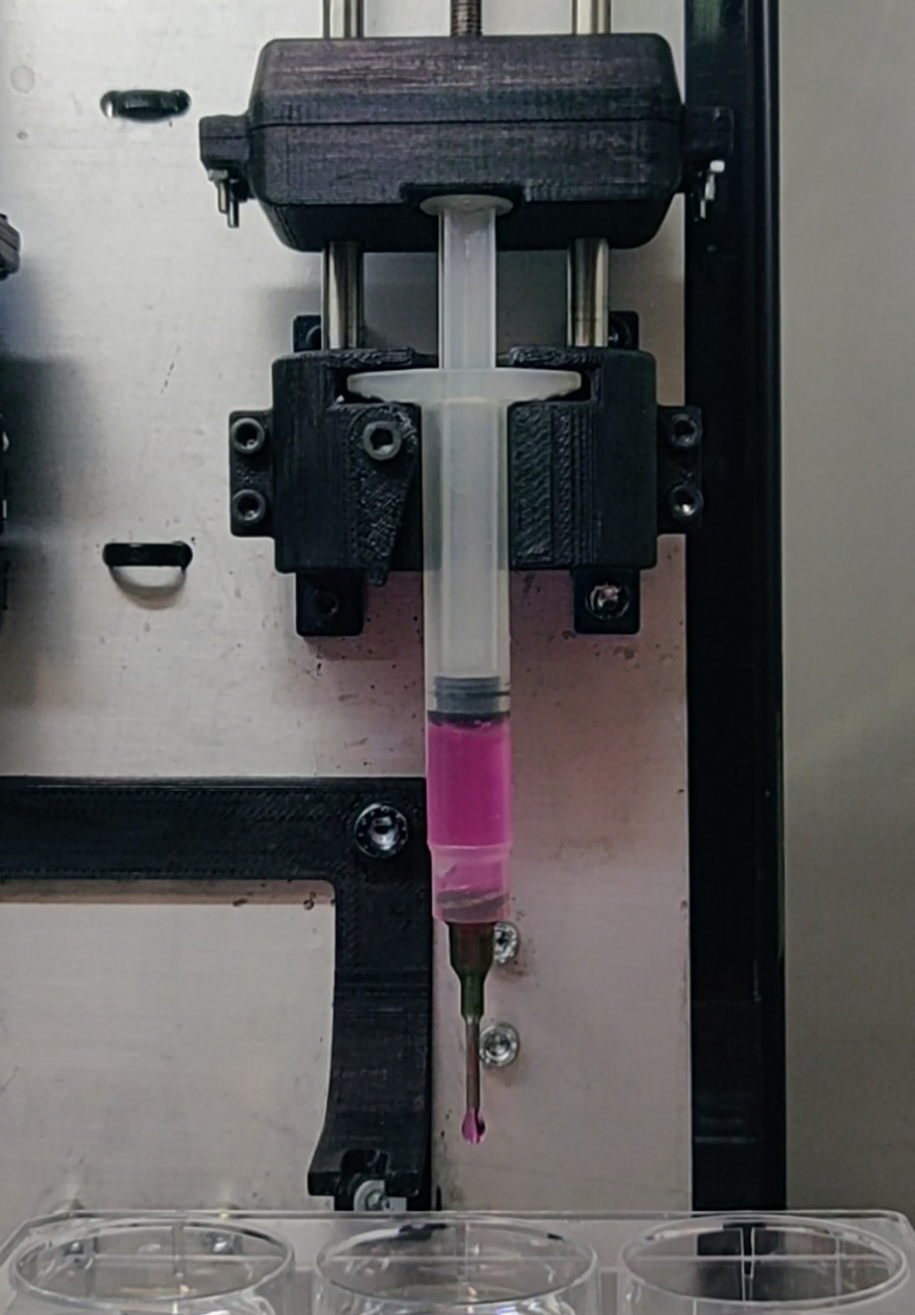
Fabrication of cell-laden spheroids using the SpHyGa bioprinting platform.^37^ The video shows the whole process performed under a laminar flow hood: first, the filling of the syringe with the cell suspension; subsequently, the drop-by-drop extrusion and release of the suspension in the cross-linking bath to form spheroidal cell-laden hydrogels (video available in the supplementary material).

Cell-laden spheroids were fabricated through the SpHyGa (spherical hydrogel generator) bioprinting platform (see supplementary material, Fig. S1).^37^ The cells were suspended at different volumetric densities, ranging from 1 × 10^12^ to 7.5 × 10^12^ cell m^−3^ (Table S2) in DMEM containing 1% w/v alginic acid and 0.004% type I collagen. The suspension was extruded as discrete droplets from the SpHyGa platform in an electrolytic bath of CaCl_2_ 0.1 M and incubated during alginate cross-linking for 15 min. The spheroids were moved to fresh DMEM and incubated overnight before testing. Details of the extrusion process are reported in the supplementary material (Sec. SM2), while the spheroid fabrication is depicted in Fig. 5 (and the associated video in the supplementary material).

### Cell viability and density assessment

Before starting each batch of experiments, at least three samples were used to evaluate cell viability and the effective cell density. In the monolayers, cells were detached from the culture surface with trypsin-EDTA (Lonza^®^, Basel, Switzerland); spheroids were left in a solution of 55 mM citric acid (Alfa Aesar^®^, Haverhill, Massachusetts, USA) and incubated until complete dissolution of the hydrogel matrix. Then, the cells were centrifuged for 5 min at 900 rpm (Centrifuge 5702, Eppendorf^®^, Hamburg, Germany) and resuspended in 200 *μ*l of DMEM.

A sample of 30 *μ*l was then mixed with the same volume of trypan blue (Thermo Fisher Scientific^®^, Walthan, Massachusetts, USA), and an automated cell counter (Countess II FL, Thermo Fisher Scientific^®^) was used to determine cell number and viability. O_2_ measurements were only performed on batches in which the percentage of viable cells was > 90%.

The cell surface density was obtained knowing the size of a 96-well bottom. The volumetric cell density was instead determined from images of the spheroids acquired to compute their volume. More specifically, construct equivalent radii were estimated as the mean of those returned by using three different measurement tools in ImageJ.^38^

### Oxygen concentration measurements

O_2_ concentration measurements were performed using commercial sensors based on O_2_ quenching (Pyroscience^®^ GmbH, Aachen, Germany). A wide variety of sensing elements with the same functioning principle are available.

For cell monolayers, a contactless O_2_ sensor spot (OXP5) attached to the bottom of a 96-well plate [Fig. 1(A)] was used to acquire the average O_2_ concentration at the cell level as a function of time. The well size was chosen to match spot dimensions, so that O_2_ concentration is averaged over the whole surface of the cell culture. Sensor spots were fixed onto the plate bottom using a silicone glue (RS Components^®^, London, UK), and optical signals were collected and transmitted to the processing unit through an optical fiber. For cell-laden spheroids, a needle probe microsensor (OXR50), consisting of a needle housing a retractable optic fiber with a 50 *μ*m sensing tip [Fig. 1(B)], was directly connected to the processing unit. For both kinds of probe, a two-point sensor calibration was carried out in exactly the same conditions as those of the corresponding experiments for establishing the level of 100% O_2_ saturation and the totally anoxic condition, using the protocol reported in Ref. 20.

For both 2D and 3D cultures, the air-medium interface was rendered O_2_-impermeable by combining the use of a thick layer of silicone oil [Agilent Technologies^®^, St Clara, California, USA—Figs. 1(A) and 1(B)] and an O_2_-consuming chemical filter (AnaeroGen, Thermo Fisher Scientific^®^). This combination was necessary because silicone oil alone—despite being advertised as O_2_ impermeable—was not sufficient to guarantee the absence of O_2_ diffusion into the culture medium in the timescales of interest. All experiments were carried out at 37 °C using a purposely designed heating system composed of a transparent chamber made up of poly-methyl-methacrylate (PMMA, Plexiglass^®^) and an electronic module implementing a PID control. The chemical filter was left in the chamber to continuously scavenge O_2_ and so maintain the boundary concentration at the air–medium interface close to zero. A water reservoir was also placed in the chamber to avoid evaporation of culture medium. Given the limited duration of measurements, the CO_2_ partial pressure was not controlled.

For monolayers, the initial condition was set to 100% O_2_ saturation by dispensing a minimum volume (20 *μ*l) of fresh DMEM onto the cells, and the acquisition started immediately. The measurement terminated after a hypoxic steady state (i.e., O_2_ saturation lower than 20%, corresponding to a concentration of 0.04 mol m^−3^)^5^ was reached, which took from half an hour to a few hours depending on the cell surface density. Sensor spots were re-used for a few cycles, after cell detachment with trypsin-EDTA and rinsing with a 70% v/v ethanol solution.

**FIG. 6.**
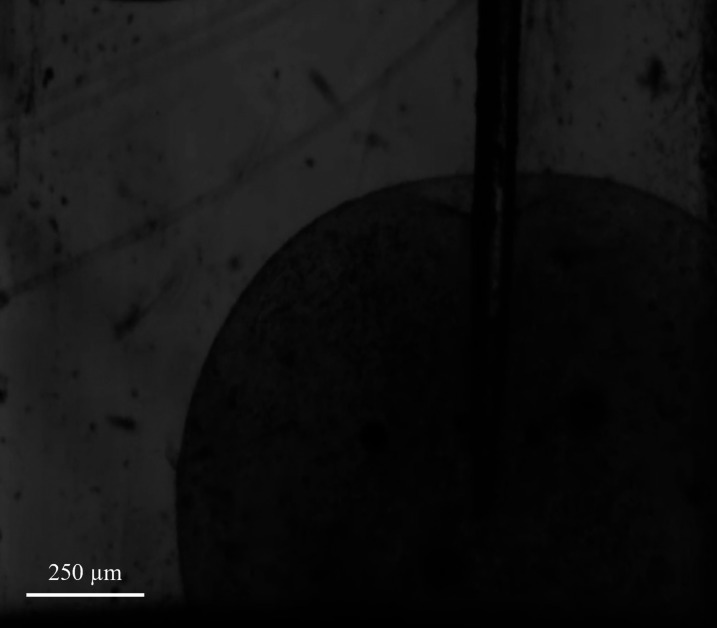
Positioning of the optical microsensor at the center of the cell-laden spheroid. The video—directly recorded by the frontal camera of the tensiometer—shows the spheroid placed within the cuvette during the insertion of the sensor tip, which is accurately moved to the desired spatial point using the micromanipulator. The shape retention of the construct after puncturing can be also noticed.

Cell-laden spheroids were placed in a transparent cuvette containing 200 *μ*l of fresh DMEM. A micro-manipulator and the camera of an optical tensiometer (Attension Theta Lite, Biolin Scientific^®^, Goteborg, Sweden) were used to accurately position the sensor tip at the center of the spheroid [Fig. 1(B)] until the stationary hypoxic condition was reached (implying measurements lasting from 10 min to an hour). Note that a single pointwise measurement over time of the local O_2_ concentration at a controlled spatial position within the spheroid was enough to identify MM parameters, because the setup geometry (i.e., culture medium height, construct diameter) is known and—as shown in Fig. 6 (and associated video in the supplementary material)—the insertion of the probe did not affect the spheroidal shape of the construct. Between consecutive acquisitions, the sensing tip of the probe was rinsed with a 70% v/v ethanol solution.

For all experiments, several replicates were performed for each cell density and dimensionality (see supplementary material, Tables S1 and S2).

### Kinetic parameter identification

**FIG. 7.**
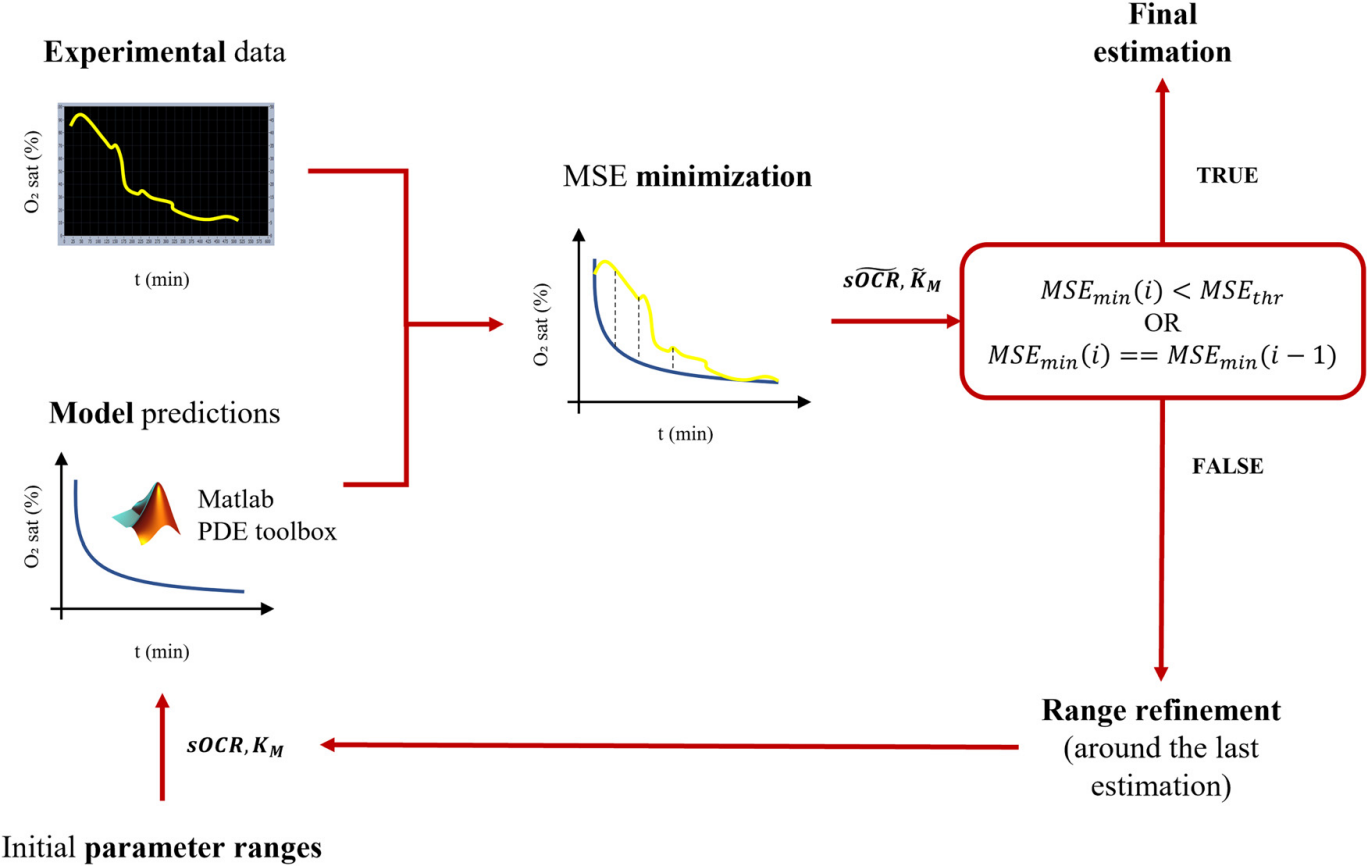
Pipeline of the multiparameter identification workflow for sOCR and $k_{M}$. For each sample, the optimal values of sOCR and $k_{M}$ are determined by iteratively simulating the O_2_ transport and consumption dynamics of the system using the PDE toolbox in MATLAB, until the MSE (mean squared error) between predicted and measured O_2_ concentration profiles is minimized.

The concentration profiles obtained were post-processed to estimate numerical values of the MM parameters through a multiparameter identification pipeline shown in Fig. 7. Basically, sOCR and $k_{M}$ were identified for each tested configuration by iteratively minimizing the mean squared error (MSE) computed between the measured O_2_ concentration profile and the one obtained solving the non-dimensional form of Eqs. (2) and (4) using the partial differential equations (PDE) toolbox in MATLAB (R2021b). All parameters as well as domain and boundary conditions used for modeling O_2_ measurements in both cases are listed in Tables I–III, respectively. For each combination of sOCR and $k_{M}$, the O_2_ concentration profile over time was extracted and rescaled from the non-dimensional concentration field returned by the PDE solver to evaluate the MSE with respect to the corresponding experimental dataset. Starting from reasonable initial ranges of the two MM parameters (Table II), the minimization problem was iteratively solved, and the ranges of parametric sweep correspondingly refined around the last estimated values. The algorithm stops and returns the final estimation of sOCR and $k_{M}$ when the MSE is below a predefined threshold of tolerance (set as 0.005 mol m^−3^, ∼ an order of magnitude lower than the assumed hypoxic threshold) or stabilizes over two consecutive iterations (Fig. 7).

**TABLE II.** Parameters used for simulating O_2_ dynamics for both dimensionalities (cell monolayers = 2D, cell-laden spheroids = 3D).

Dimensionality	Parameter	Numerical value	Description
2D	h	625 *μ*m	Medium height
3D	L	5 mm	Cuvette length
3D	W	10 mm	Cuvette depth
3D	H	$\frac{V_{\text{med}}+\frac{4}{3}\pi {r_{\text{sph}}}^{3}}{LW}$	Medium height
2D	$D_{w}$	3 × 10^–9^ m^2^ s^−1^ (Ref. 5)	O_2_ diffusion coefficient in water at 37 °C
3D	$D_{h}$	1.1 × 10^–9^ m^2^ s^−1^ (Ref. 20)^a^	O_2_ diffusion coefficient in cross-linked hydrogels at 37 °C
Both	$C_{0}$	0.2 mol m^−3^ (Ref. 4)	Maximum [O_2_]
2D	$\rho_{s}$	See Table S1	Surface cell density
3D	$\rho_{v}$	See Table S2	Volumetric cell density
Both	sOCR	(10^–17^; 10^–16^) mol cell^−1^ s^−1^ (Refs. 4 and 39)	Initial range of parametric sweep
Both	$k_{M}$	(10^–3^; 10^–2^) mol m^−3^ (Refs. 4, 18, and 39)	Initial range of parametric sweep

^a^ Median of values identified in Ref. 20, in which gels and cell densities were similar to those used here.

**TABLE III.** Domain conditions implemented for simulating O_2_ dynamics for both dimensionalities (cell monolayers = 2D, cell-laden spheroids = 3D).

Dimensionality	Domain	Condition	Description
2D	Culture medium	Eq. (4)	1D diffusive transport
3D	Culture medium, spheroid	Eq. (2)	3D diffusive transport and consumption
3D	Culture medium	R=0	No consumption
2D	Spheroid	Eq. (3)	MM consumption
Both	All	$C\left(z,0\right)=C_{0}$, $C\left(x,y,z,0\right)=C_{0}$	Initial condition

To assess whether a global fitting for the simultaneous determination of both MM parameters improves the quality of results, a (mono)parameter identification was also run; $k_{M}$ was set at literature values to estimate sOCR and vice versa. Section SM4 in the supplementary material reports further details and data on the multiparameter identification procedure.

### Staining and imaging

For each cell density, three samples were fixed with a 4% w/v paraformaldehyde (PFA) solution and then stained with phalloidin red (Phalloidin-iFluor 647, Abcam^®^, Cambridge, UK) and 4′, 6-diamidino-2-phenylindole (DAPI, Sigma-Aldrich^®^) using the manufacturers' protocols (supplementary material, Fig. S2). Constructs were left in PBS 1× and stored in the dark at 4 °C until image acquisition. Imaging was performed using a Nikon A1 confocal laser microscope (Nikon^®^, Tokyo, Japan), equipped with a 10× objective (pixel size: 0.31 *μ*m).

### Proximity index estimation

To identify a common metric to describe the extent of cell packing independent of the culture dimensionality (i.e., for both 2D and 3D cultures), confocal images were post-processed using *ad hoc* routines developed in MATLAB. Specifically, the blue channel was segmented to highlight cell nuclei (see the supplementary material, Fig. S2) and identify their centroid coordinates. Then, the weighted mean $p_{i}$ (*μ*m^−1^) of the inverse distance of each centroid from all the other nuclei within the imaged region was computed. Sholl analysis^40,41^ allowed the estimation of the number of cell nuclei $n_{ij}$ at a distance $d_{j}$ from the *i*th cell, representing the *j*th weight for averaging inverse distances from that cell. Thus, $p_{i}$ is given by the following equation:

$$p_{i}=\frac{1}{N_{d}}\sum_{j=1}^{N_{d}}\frac{n_{ij}}{d_{j}},$$ (5)

where $N_{d}$ is the number of distances considered for the Sholl analysis to encompass the range [5.0; 179.2] *μ*m with a step of 10 *μ*m. The lower limit of the range corresponds to the radius of the smallest nucleus detected, while the higher limit is set by the size of the stack. Note that, for consistency, the total number of cells in the stack is $N_{\text{cell}}=\sum_{j=1}^{N_{d}}n_{ij}$. The proximity index PI (*μ*m^−1^) was then defined as in the following equation, which is the average value of $p_{i}$ over all cells detected in the sample:

$$PI=\frac{1}{N_{\text{cell}}}\sum_{i=1}^{N_{\text{cell}}}p_{i}.$$ (6)

For 3D spheroids, Eqs. (5) and (6) were used to compute PI values of both the whole construct and 10 *μ*m-thick cross-sectional regions across its volume, obtained using different slicing orientations. The slice thickness corresponds to the diameter of the biggest nucleus detected. The PI accounts for both cell density and dimensionality and can thus be considered as a unique metric for comparing 2D and 3D cultures in terms of cell packing.

We also estimated the average sphericity S (a.u.) of cell nuclei within each image stack using the following equation:^42^

$$S=\frac{1}{N_{\text{cell}}}\sum_{i=1}^{N_{\text{cell}}}\left(\frac{\sqrt[{3}]{36\pi {V_{i}}^{2}}}{A_{i}}\right),$$ (7)

where $A_{i}$ (m^2^) and $V_{i}$ (m^3^) are, respectively, the surface area and the volume of the *i*th cell detected. They were automatically determined using the MATLAB's built-in connected component analysis.

### Statistical analysis

Statistical analyses were performed in GraphPad Prism (v7) by means of a non-parametric Kruskal–Wallis test and pairwise *post hoc* Dunn's multiple comparisons for PI and sphericity. The Kruskal–Wallis test and related multiple comparisons, as well as non-linear correlation (i.e., non-parametric Spearman coefficient) with respect to (*i*) the surface or volumetric cell density and/or (*ii*) the sample PI were used to analyze the dataset of identified sOCR and $k_{M}$ values. Nonlinear correlation with PI was also tested for φ.

Note that, given the limited number of samples used, all quantities were expressed as median ± range, and only non-parametric tests were performed.

## SUPPLEMENTARY MATERIAL

See the supplementary material that contains supporting technical information on the cell maintenance, fabrication of cell-laden-spheroids, multiparameter identification and statistical analysis. “Figures 1 and 3” of the supplementary material are videos of, respectively, the bioprinting process of spheroids and the microsensor positioning recorded via the frontal camera of the tensiometer.

## Data Availability

The data that support the findings of this study are available from the corresponding author upon reasonable request.
